# Guishaozichuan granules can attenuate asthma in rats *via* the MUC5AC/EGFR signaling pathway

**DOI:** 10.3389/fphar.2022.1011751

**Published:** 2023-01-09

**Authors:** Qinqin Gao, Chenran Feng, Qi Shi, Qingling Wang, Zitong Ding, Huilun Chu, Deming Kong, Xingbin Yin, Jian Ni, Wenyan Sun, Youlin Li

**Affiliations:** ^1^ Department of Pharmacology of Chinese Materia Medica, School of Chinese Materia Medica, Beijing University of Chinese Medicine, Beijing, China; ^2^ The 2nd Pulmonary Department of TCM, Beijing Key Laboratory (No. BZ0321), The Key Institute of State Administration of Traditional Chinese Medicine (Pneumonopathy Chronic Cough and Dyspnea), China-Japan Friendship Hospital, Beijing, China

**Keywords:** guishaozichuan granules, bronchial asthma, cytokines, MUC5AC/EGFR signal pathway, lung function

## Abstract

**Background:** Guishaozichuan (GSZC) granules are a traditional Chinese medicine formulation created by Professor Li (Chinese–Japanese Friendship Hospital, Beijing, China) we studied the effect of GSZC granules in rats suffering from asthma.

**Methods:** Specific pathogen-free Sprague–Dawley rats were divided randomly into seven groups. Ovalbumin (OVA) and Al (OH)_3_ gel were used to create an asthma model. On day 1, rats were injected with OVA (10 mg) and an Al(OH)_3_ gel suspension (100 mg). One week later, rats were sensitized again. On day 15, rats were given aerosolized OVA (1%) for 30 min/day for 10 days. Gastric administration of OVA was 1 h before nebulization. At 24 h after the last stimulation, changes in airway resistance (R_I_) and dynamic compliance (C_dyn_) in rat lungs were measured after challenge with methacholine at increasing concentrations. The contents of immunoglobulin (Ig)E, interleukin (IL)-4, IL-5, IL-13, and IL-17 in serum were measured by enzyme-linked immunosorbent assays. The percentage of eosinophils (EOS) and the white blood cell (WBC) count in bronchoalveolar lavage fluid (BALF) were counted under an optical microscope. Pathologic alterations in lung tissue were evaluated by optical microscopy, and lung injury score calculated. Expression of mucin 5AC, oligomeric mucus/gel-forming (MUC5AC) and epidermal growth factor receptor (EGFR) in lung tissue was measured by immunohistochemistry. mRNA expression of MUC5AC and EGFR in lung tissue was measured by real-time reverse transcription-quantitative polymerase chain reaction (RT-qPCR).

**Results:** GSZC granules reduced R_I_ markedly and improved C_dyn_, decreased serum levels of IgE, IL-4, IL-5, IL-13, IL-17, %EOS and the WBC count in BALF. GSZC granules alleviated lung-tissue damage, diminished the Inflammation Score, and reduced mRNA and protein expression of MUC5AC and EGFR in lung tissue.

**Conclusion:** GSZC granules could improve bronchial hyperresponsiveness, bronchial inflammation, and histopathologic damage in the lungs of rats suffering from asthma. This phenomenon may be related to its regulation of cytokine levels and the MUC5AC/EGFR signaling pathway.

## 1 Introduction

Asthma is a recurrent chronic respiratory disease (Zhou, 2015). Common clinical symptoms include exertional breathlessness with coughing, dyspnea, and chest tightness. Patients with severe asthma are forced to sit down and concentrate on breathing. Asthma symptoms can occur within a few minutes. After taking immunosuppressants (e.g., glucocorticoids), symptoms are relieved in most patients suffering from asthma. Approximately 5%–10% of patients find controlling an asthma attack to be difficult (Xu, 2016). Asthma is a chronic, non-specific inflammatory disease of the respiratory tract. Different types of inflammatory cells and inflammatory mediators are involved in asthma. Asthma patients are prone to develop chronic bronchitis and cor pulmonale, which can threaten life (Wu, 2017). The Chinese Center for Disease Control and Prevention discovered that asthma prevalence among Chinese adults (age >18 years) in 2010 was 1.09% (Cong et al., 2015), and that severe asthma affected 5%–10% of all asthma patients (Peng et al., 2018), indicating that there are deficiencies in the prevention and treatment of asthma in China. Therefore, in-depth study of the pathogenesis and treatment of asthma is important.

Traditional Chinese medicine (TCM) formulations have been used for the treatment of asthma for centuries. Intervention with TCM formulations can nourish qi and the kidney, supplement the lung, tonify the spleen, remove blood stasis and phlegm, and strengthen the body. Studies have shown that many Chinese herbs (including compound prescriptions, single herbs, and monomer) can reduce the inflammation, hyperresponsiveness, and mucus secretion observed in the airways of asthma patients. This phenomenon may be associated with immune regulation by T cells, including reducing the response of T helper type 2 (Th2) or Th17 cells, enhancing the function of T regulatory cells (Li et al., 2017), and downregulating expression of mucin 5AC, oligomeric mucus/gel-forming (MUC5AC).

A formulation called Guishaozichuan (GSZC) granules was created by Professor Li of the China–Japan Friendship Hospital (Beijing, China) for asthma treatment induced by a deficiency of the lung‐spleen and impairment of dispersion and descending of the lungs. GSZC granules comprise *Astragalus membranaceus* (Fisch.) Bge, *Sinapis alba* L, *Atractylodes macrocephala* Koidz, *Zingiber officinale* Rosc, *Perilla Frutescens* (L.) Britt, *Paeonia lactiflora* Pall, *Magnolia officinalis* Rehd. et Wils, *Cinnamomum cassia* Presl, *Schisandra chinensis* (Turcz.) Baill. and *Glycyrrhiza uralensis* Fisch. (www.theplantlist.org). GSZC granules have been used to strengthen the spleen, benefit the lung, diminish qi, and they also have anti-asthma actions.

Among the herbs contained in GSZC granules, *Astragalus membranaceus* (Fisch.) Bge. invigorates the spleen, benefits the lungs, and tonifies body’s vital energy (known as qi); *Cinnamomum cassia* Presl. can warm meridians, assist positive (known as yang), and turn qi. A combination of these two herbs can strengthen the spleen and benefit the lung, and they are used as “monarch” drugs. *Atractylodes macrocephala* Koidz. invigorates the spleen and qi, dries dampness and diuresis, stops sweating, cultivates soil (known as Tu, in the field of Chinese medicine it represents the spleen), and eliminates the source of phlegm; *Perilla Frutescens* (L.) Britt, descending qi, eliminates phlegm and relieves asthma. A combination of these two herbs can strengthen the spleen, eliminate phlegm, cultivate Tu, generate gold (known as Jin, in the field of Chinese medicine it represents the lung), reduce qi, and relieve asthma. These two herbs are called “minister drugs”. *Paeonia lactiflora* Pall. can calm the liver, relieve pain, nourish the blood, regulate menstruation, collect negative (known as Yin), and stop sweating; *Schisandra chinensis* (Turcz.) Baill. is an astringent, and tonifies qi, promotes fluids, tonifies the kidneys, and calms the heart. These two herbs are used as adjuvant drugs to help monarch drugs and minister drugs. *Glycyrrhiza uralensis* Fisch. Is a “courier” drug that can invigorate the spleen and qi, clear heat, detoxify, eliminate phlegm, alleviate cough, and reconcile various drugs.

GSZC granules work by the principle of “wenrun xinjin peiben” (moisten the lung and cultivate the spleen). Use of GSZC granules leads to sweetness and warmth to replenish qi, spice and sweetness to turn Yang, sour and sweetness to turn Yin, and a combination of Yin, and Yang. GSZC granules take the lung and spleen to be the core to allow overall syndrome differentiation of viscera, seek the cause of the disease, as well as warm Yang, strengthen the spleen and lungs, reduce qi, and relieve asthma.

GSZC granules have been shown to significantly improve the clinical symptoms and signs of asthma patients, with a high safety and no obvious adverse reaction. (Zhang and Li, 2013; Si et al., 2019). Ding and colleagues showed that GSZC granules exerted protective effects against bronchial hyperresponsiveness, the inflammatory reaction, and damage to lung tissue in an ovalbumin (OVA)-induced asthma model in mice (Ding et al., 2020). GSZC granules also have anti-asthma, antitussive, and expectorant effects (Shi et al., 2020).

We created an OVA-induced asthma model in rats to observe the effect of GSZC granules on airway reactivity. We measured airway resistance (R_I_) and dynamic compliance (C_dyn_). We noted the effect of GSZC granules on airway inflammation by measuring levels of immunoglobulin (Ig)E, proinflammatory cytokines, the percentage of eosinophils (EOS), the white blood cell (WBC) count, and lung histopathology. We observed the effect of GSZC granules on MUC5AC/EGFR signal pathway. Our study may provide a reference for the clinical application of GSZC granules.

## 2 Experimental

### 2.1 Materials

OVA was purchased from MilliporeSigma (SLBV7493; Burlington, MA, United States). Al(OH)_3_ was obtained from Pierce (SLBV7493; Rockford, IL, United States). Acetylcholine was sourced from BioRuler (r621712; Dansbury, CT, United States). Phosphate-buffered saline (PBS) was purchased from Jiangsu Keygen (20180228; Jiangsu, China). Pentobarbital sodium was obtained from MilliporeSigma. Neutral buffered formalin solution (10%) was sourced from Beijing Yili Fine Chemicals (Beijing, China). Dexamethasone acetate was from Tianjin Lisheng Pharmaceuticals (1705021; Tianjin, China).

Yupingfeng granules (one Chinese patent drug) was purchased from Sinopharm (Z10930036; Beijing, China). Liujunzi pills (another Chinese patent drug) was purchased from Jilin Zixin (Z22024784; Jilin, China) GSZC granules (1 g of extract was equivalent to 7.35 g of crude drug) was provided by the Department of Pharmacy of Beijing University of Chinese Medicine (Beijing, China). Three batches of GSZC granules (20,180,101, 20,180,102, 20,180,103) were obtained from the Department of Pharmacy of Beijing University of Chinese Medicine (Beijing, China). Alcohol (75%) was purchased from Shandong Mingde (Shandong, China).

Enzyme-linked immunosorbent assay (ELISA) kits for IgE (20190719 and 60139R), interleukin (IL)-4 (20190719 and 60021R), IL-5 (20190719 and 60022R), IL-13 (20190719 and 60029R), and IL-17 (20190719 and 60032R) were purchased from Beijing Rigorbio Science Development (Beijing, China). A Reverse Transcription Kit was obtained from Promega (Fitchburg, WI, United States). SYBR^®^ Premix Ex Taq II was sourced from Takara Biotechnology (RR820A; Shiga, Japan). TRIzol^®^ Reagent was obtained from Invitrogen (Carlsbad, CA, United States). Epidermal growth factor receptor (EGFR) antibody was from Proteintech (18986-1-AP; Chicago, IL, United States). MUC5AC antibody was purchased from Boster Group (20180824; Wuhan, China). Astragaloside (110781–201515) and paeoniflorin (110736–201640) were purchased from National Institutes for Food and Drug Control (Beijing, China). Acetonitrile and formic acid, both of high-performance liquid chromatography (HPLC) grade, were obtained from MilliporeSigma. All other reagents were of analytical grade and sourced from Beijing Chemical Factory (Beijing, China). Water was purified using a Milli-Q^®^ system (Millipore, Bedford, MA, United States).

### 2.2 Preparation and quantitative analysis of an extract of GSZC granules

*Astragalus membranaceus* (Fisch.) Bge. is the herb with the highest content in GSZC granules and contains astragaloside. *Paeonia lactiflora* Pall. is the herb with the second highest content in GSZC granules, and contains paeoniflorin. Astragaloside and paeoniflorin were selected as the indicators for quantitative analyses of GSZC granules.

An HPLC method was established to measure the content of astragaloside and paeoniflorin. First, a sample solution of astragaloside was prepared. Briefly, a standard of astragaloside (4 g) was weighed accurately, placed in a conical bottle with a plug, and extracted ultrasonically with N-butanol (60 mL) for 30 min. After cooling at room temperature, the mixture was filtered. The filtrate was concentrated to dryness. The residue was dissolved in N-butanol, extracted four times with water-saturated N-butanol (40 mL) by shaking, and combined with N-butanol solution. The solution was washed twice with ammonia test solution (50 mL each time) and the ammonia test solution discarded. Then, the N-butanol solution was evaporated. The obtained residue was dissolved in an appropriate amount of methanol, transferred to a 5 mL volumetric flask, made up to 5 mL with methanol, and shaken well. The resulting solution was used as the test solution.

A standard of paeoniflorin (.5 g) was weighed precisely. The fine powder was placed in a conical bottle with a plug and extracted ultrasonically with methanol (100 mL) for 45 min. After cooling at room temperature, the loss in weight was made up with methanol, and the solution shaken well. This filtrate solution was used as the test solution.

Astragaloside and paeoniflorin (the two main pharmacologically active constituents of GSZC granules) were assayed with 1100 (Agilent Technologies, Santa Clara, CA, United States) and LC-20AT (Shimadzu Technologies, Tokyo, Japan) HPLC systems. The former was equipped with a vacuum degasser, quaternary pump, diode array detector, autosampler, and a column temperature controller. The LC-20AT system consisted of a SIL-20AC autosampler and SPD-20A PDA detector that used LabSolutions (Weiswampach, Luxembourg) software.

**FIGURE 1:**
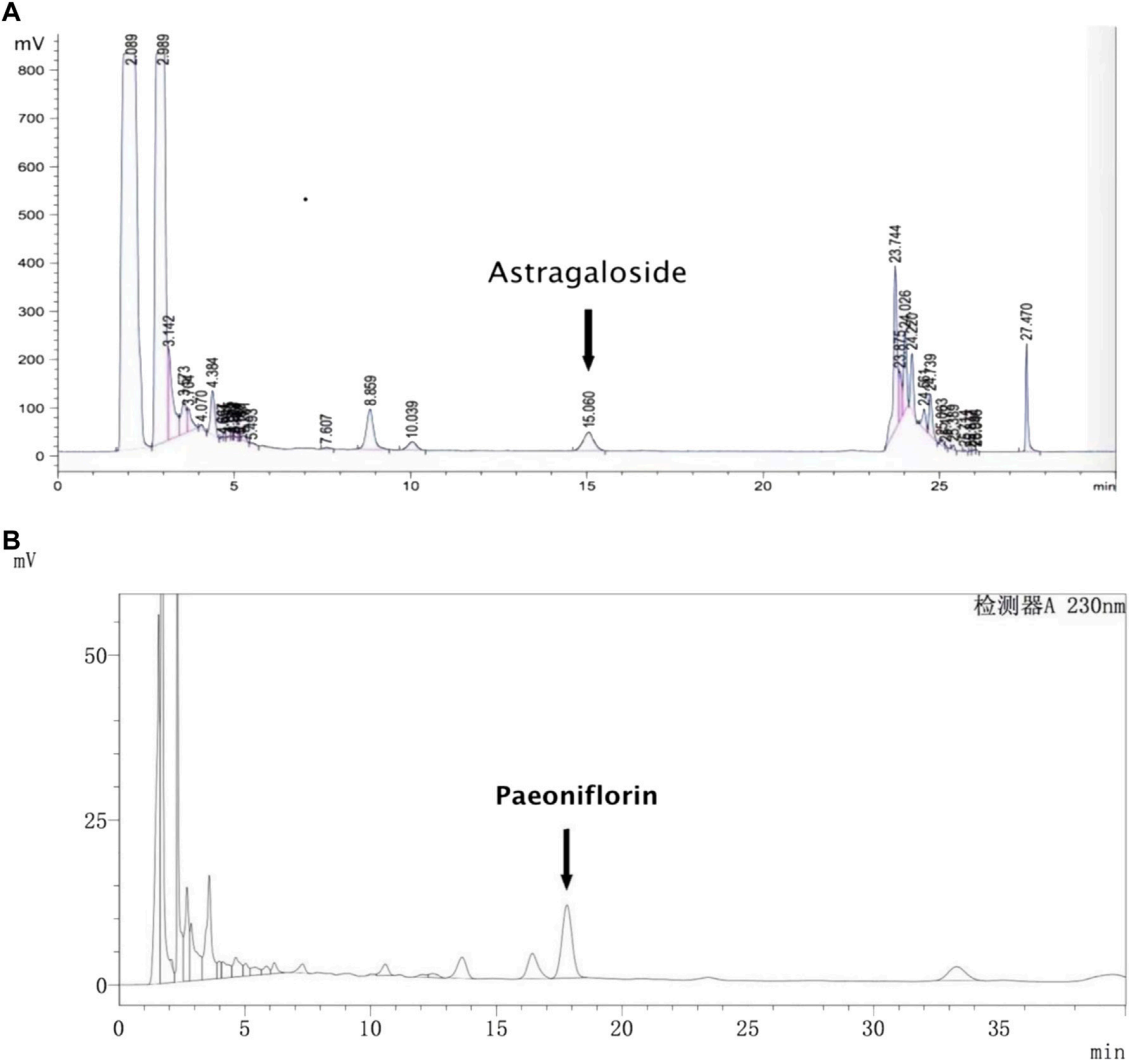
HPLC chromatogram of astragaloside **(A)** and paeoniflorin **(B)**.

The analysis of astragaloside was undertaken on a Zorbax SB-C18 column (250 mm × 4.6 mm, 5 μm; Agilent Technologies) at 30°C using a mobile phase comprising acetonitrile and water (32:68). The settings for the evaporative light-scattering detector were: flow rate of the carrier gas (compressed air) = 2.7 L/min; temperature of the drift tube = 100°C; injection volume = 20 μL. The analysis of paeoniflorin was also carried out on a Zorbax SB-C18 column (4.6 mm × 250 mm, 5 μm; Waters, Milford, MA, United States ) at 30°C using a mobile phase comprising acetonitrile and .1% phosphoric acid (11:89). The flow rate was 1.0 L/min, the wavelength was set at 230 nm, and the injection volume was 10 μL. Under the conditions described above, HPLC peaks could be separated clearly (Figure 1). The contents of three batches of samples were determined using the method described above. The average content of astragaloside and paeoniflorin was .35 mg/g and 4.30 mg/g, respectively.

### 2.3 Animal model of asthma

The experimental protocol was approved (1802010) by the Experimental Animal Welfare Ethics Committee of China–Japan Friendship Hospital. Experiments were conducted in accordance with the *Guide for the Care and Use of Laboratory Animals* (National Institutes of Health, Bethesda, MD, United States).

Seventy specific pathogen-free Sprague–Dawley (8–9 weeks) rats of either sex were obtained from SiPeiFu Beijing Biotechnology (certificate number: SCXK (Jing) 2016-0002; Beijing, China). Under pathogen-free conditions, rats were kept in a cage at a constant temperature (23°C ± 2°C) and subjected to a 12 h day‐night cycle (lights on at 8 a.m. and lights off at 8 p.m.) with *ad libitum* access to food and water. The humidity level ranged between 40%–55%.

After feeding for 1 week, rats were sensitized using 1 mL of OVA (10 mg) plus Al(OH)_3_ (100 mg) as a suspension (intraperitoneal injection of .5 mL, groin injection of .5 mL) on day 1. Sensitization was conducted again 1 week later; the normal group was injected with the same dose of physiologic (.9%) saline. On day 15, all rats except the normal group were challenged with an aerosol of 1% OVA for 30 min for a total of 10 days using a 402B ultrasonic nebulizer (Jiangsu Yuyue Medical Equipment and Supply, Jiangsu, China). The normal group was given .9% saline instead.

### 2.4 Drug administration and animal grouping

The 70 rats of either sex were divided randomly into seven groups of 10: Normal, model, GSZC granules low-dose (.67 g/kg; GSZC-L), GSZC granules moderate-dose (1.34 g/kg; GSZC-M), GSZC granules high-dose (2.68 g/kg; GSZC-H), positive drug (dexamethasone acetate (DEX), .0006 g/kg), and TCM-positive drug (Yupingfeng granules (1.51 g/kg) + Liujunzi pills (1.8 g/kg), YPF + LJZ).

The dose of drug administration was based on the body surface area of the rat and normal clinical use of the crude drug (Xu et al., 2002). Considering the moderate dose to be an equivalent dose, the low dose was equal to .5 times the moderate dose, and the high dose was equal to two times the moderate dose. Dexamethasone acetate, Yupingfeng granules, and Liujunzi pills were administered as an equivalent dose.

15 days after the model had been established, gavage administration in the dosing group was performed 1 h before challenged with an aerosol of 1% OVA for 10 days. Simultaneously, the normal group administered the same amount of .9% saline. Dexamethasone is a synthetic glucocorticoid. It is one of the most potent drugs used to treat asthma, and was used as the positive control drug (Dong et al., 2021). Yupingfeng granules can tonify the lung and strengthen qi, and are used to treat asthma. Liujunzi pills invigorate the spleen and eliminate phlegm to treat deficiency of the spleen that leads to asthma. The combination of Yupingfeng granules and Liujunzi pills has similar effects to those of GSZC granules (invigorate the spleen, benefit the lung, regulate qi, and eliminate phlegm (Liu Y. et al., 2019), and was used as a positive control.

### 2.5 Lung-function test

At 24 h after the final aerosol challenge, rats were anesthetized with 2% pentobarbital (60 mg/kg, i. p.). A skin incision was made on the ventral side of the neck. The trachea was exposed, opened, and tracheal intubation initiated. Rats were placed in a closed plethysmography box of a pulmonary function testing system (Buxco, Wilmington, NI, United States) and artificial ventilation started. After R_I_ had returned to the baseline level, we recorded the responses 3 min after inhalation of aerosolized methacholine (0, 3.125, 12.25, or 25 mg/mL) for 30 s. The airway hyperresponsiveness of rats was assessed as described previously by measuring changes in R_I_ and C_dyn_ (Zhang and Li, 2013).

### 2.6 Measurement of levels of IgE, IL-4, IL-5, IL-13, and IL-17

Blood was sampled from the abdominal aorta. It was centrifuged at 3,000 ×*g* for 10 min at room temperature to obtain serum. Serum levels of IgE, IL-4, IL-5, IL-13, and IL-17 were measured using ELISA kits.

### 2.7 Cell counting in bronchoalveolar fluid (BALF)

We injected 2 mL, 2 mL, and 1 mL of .9% saline, respectively, slowly into the trachea. After each injection, BALF was aspirated gently back into the same syringe. BALF was centrifuged at 1,500 rpm for 10 min at room temperature and the supernatant obtained. Then, we added PBS (.2 mL) and mixed the solution, and stained with picrosirius red to count the number of EOS and WBCs. Cells were observed under a microscope (BX51; Olympus, Tokyo, Japan).

### 2.8 Histology

Rats were killed by bloodletting. The chest cavity was opened, and the lower lobe of the right lung was ligated and excised. After rinsing with .9% saline and drying with filter paper, samples of lung tissue were fixed rapidly in 10% neutral-buffered formalin. Samples of lung tissue were passed through a graded series of alcohol solutions and dehydrated. After immersion in xylene, paraffin embedding, and sectioning, hematoxylin and eosin (H&E) staining was undertaken. Six rats from each group were selected randomly. Observe the histopathology of the lung under a microscope (BX51; Olympus, Tokyo, Japan) for the following conditions to evaluate the pathologic changes and the degree of light to heavy lesions, mainly including: Edema, hyperemia and inflammatory cell infiltration in the alveolar wall, exudation of inflammatory cells and edema fluid in the alveoli; necrotic degeneration of bronchial epithelial cells, exudate in the lumen; peritube inflammatory cell infiltration. According to the degree of light to heavy lesions, small amount or no lesion was considered as negative and given a score of 0; mild or small was scored as 1; moderate or medium was scored as 2; severe was scored as 3; very heavy was scored as 4.

### 2.9 Immunohistochemical analyses to measure protein expression of MUC5AC and EGFR in lung tissue

Lung specimens were fixed in 10% formalin, dehydrated, paraffin-embedded, and sectioned. These steps were carried out according to the instructions of the antibody kit. Sections were dewaxed, hydrated, and underwent high-pressure antigen repair. Sections were washed twice (3 min each time) in PBS, then blocked for 10 min at room temperature. Subsequently, lung sections were washed thrice (3 min each time) with distilled water, then washed twice (3 min each time) with PBS, primary antibody was added dropwise, and blocked for 1 h at 37°C. Sections were washed twice (3 min each time) in PBS, secondary antibody was added dropwise, followed by blockade for 20 min at 20°C–37°C, and washing thrice (2 min each time) in PBS. 3,3′-diaminobenzidine was added to sections, and distilled water used to stop color development. Hematoxylin counterstaining was performed for 3 min and differentiation was performed using 1% hydrochloric alcohol. Finally, after dehydration and permeabilization, sections were sealed and photographed under a microscope. Images were quantified using Image Pro Plus^®^ (Media Cybernetics, San Diego, CA, United States). All fields of view were observed under 200× magnification using a microscope (BX51; Olympus, Tokyo, Japan). We recorded the value for integrated optical density of the brown–yellow area in the image calculated by Image Pro Plus, and then calculated the average optical density.

### 2.10 Real-time reverse transcription-quantitative polymerase chain reaction (RT-qPCR)

Messenger (m) RNA expression of MUC5AC and EGFR was measured by RT-qPCR using an ABI7500 instrument (Applied Biosystems, Foster City, CA, United States). Total RNA was extracted from lung tissue (100 mg) with TRIzol Reagent. The concentration and purity of RNA were determined. Complementary (c)DNA synthesis was done using a cDNA reverse transcription kit. Primers were designed using cDNA as the template. The primer sequences we used (forward and reverse, respectively) were: 5′-CAA​GCA​AGG​CCG​TGT​TGA​C-3′ and 3′-CCA​TGG​GCA​TGG​AGG​TTC​TC-5′ for MUC5AC; 5′-GCC​ACC​AAG​ACA​GGC​GAC-3′ and 3′-AGT​AGC​TTG​GTT​CTC​GCA​GT-5′ for EGFR; 5′-CAC​GCC​TAC​AGA​TCC​CAC​AG-3′ and 3′-TCT​GAG​CCT​CGT​CAC​CTA​CA-5′ for actin. RT-qPCR was undertaken using SYBR Premix Ex Taq™ II. Quantitative data are expressed as relative expression according to the formula 2^−△△ct^.

### 2.11 Statistical analyses

Data are the mean ± standard deviation. Statistical analyses were carried using SAS 9.2 (www.sas.com). For data with a normal distribution and equal variance, differences among multiple groups were assessed using one-way analysis of variance followed by the *q*-test. If the data did not have a normal distribution or the variance was not uniform, a non-parametric (Kruskal–Wallis) test was used to analyze differences between groups. *p* < .05 or *p* < .01 was considered significant.

## 3 Results

### 3.1 Effect of GSZC granules on airway responsiveness in a rat model of asthma

#### 3.1.1 Effect of GSZC granules on R_I_ in a rat model of asthma

**FIGURE 2:**
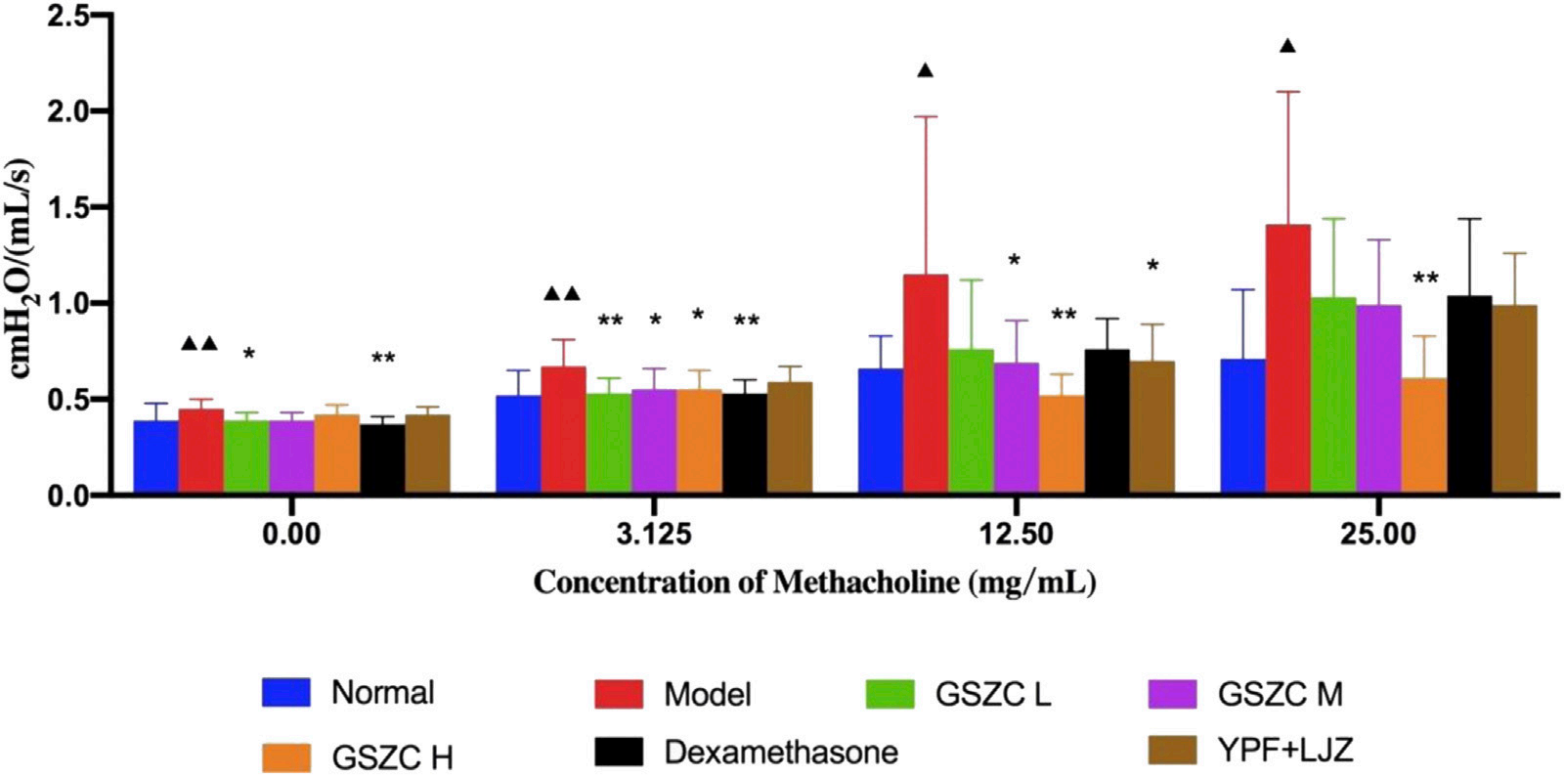
Effects of GSZC granules on airway resistance in rats suffering from asthma. Values are the mean ± SD, n = 10 for each group. ^▲^ *p* < .05, ^▲▲^ *p* < .01 *versus* the normal group, ^*^ *p* < .05, ^**^ *p* < .01 *versus* the model group.

With an increase in the methacholine dose, R_I_ in each group increased concomitantly. R_I_ was increased significantly (*p* < .05 or *p* < .01) at methacholine concentrations of 0, 3.125, 12.5, and 25 mg/mL in the model group compared with that in the normal group. R_I_ was reduced significantly (*p* < .05 or *p* < .01) at methacholine concentrations of 0 and 3.125 mg/mL in the GSZC-L group and DEX group compared with that in the model group. R_I_ was reduced significantly (*p* < .05) at methacholine concentrations of 3.125 and 12.5 mg/mL in the GSZC-M group compared with that in the model group. There was a significant reduction (*p* < .05 or *p* < .01) in R_I_ at methacholine concentrations of 3.125, 12.5 and 25 mg/mL in the GSZC-H group compared with that in the model group. R_I_ showed a difference in the YPF + LJZ group (*p* < .05) compared with that in the model group at a methacholine concentration of 12.5 mg/mL (Figure 2).

#### 3.1.2 Effect of GSZC granules on C_dyn_ in a rat model of asthma

**FIGURE 3:**
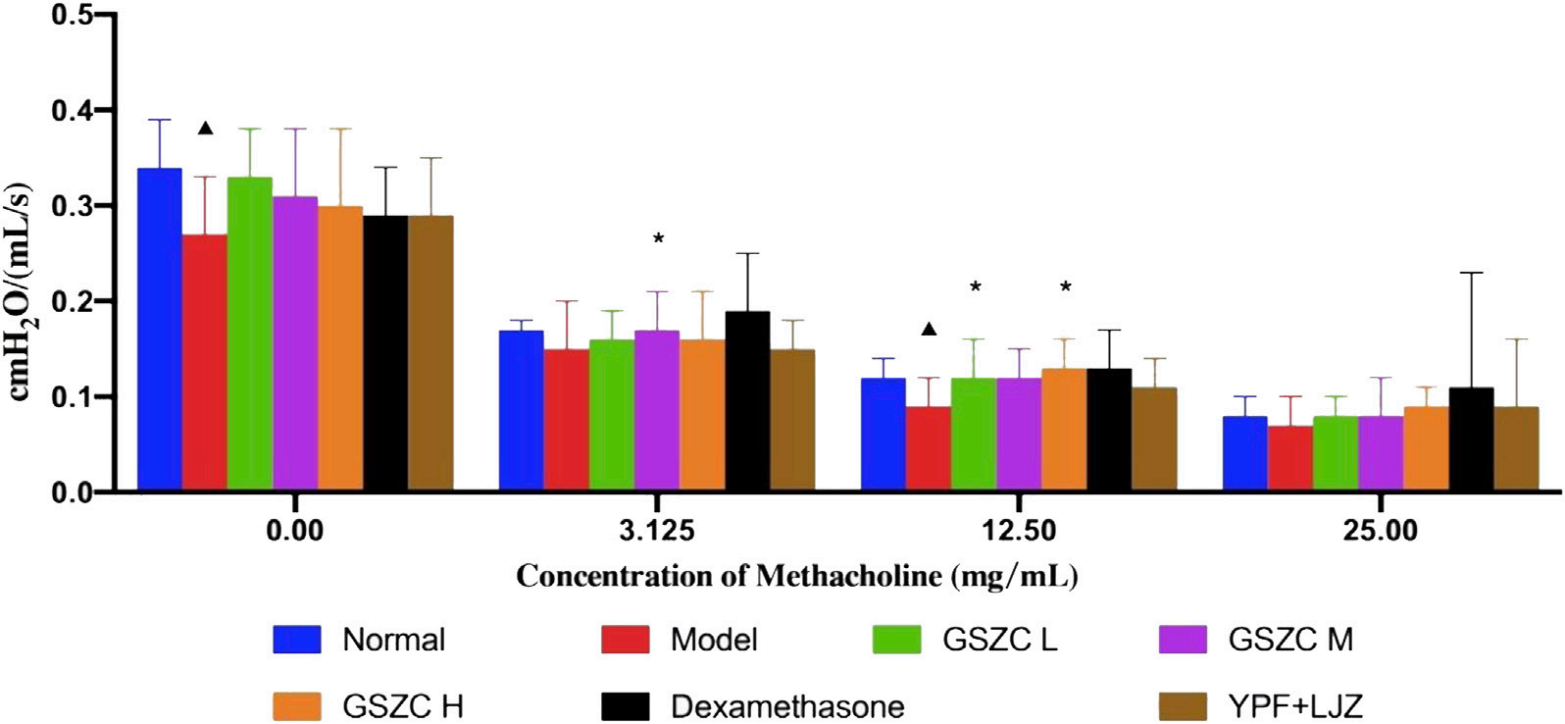
Effects of GSZC granules on airway compliance in rats suffering from asthma. Values are the mean ± SD, n = 10 for each group. ^▲^ *p* < .05 *versus* the normal group, ^*^ *p* < .05 *versus* the model group.

C_dyn_ was reduced significantly (*p* < .05) at methacholine concentrations of 0 and 12.5 mg/mL in the model group compared with that in the normal group. C_dyn_ was increased significantly (*p* < .05) at a methacholine concentration of 12.5 mg/mL in the GSZC-H group compared with that in the model group. We noted a significant (*p* < .05) increase in C_dyn_ at methacholine concentrations of 3.125 and 12.5 mg/mL in the DEX group compared with that in the model group (Figure 3).

### 3.2 Effects of GSZC granules on serum levels of IgE, IL-4, IL-5, IL-13, and IL-17 in a rat model of asthma

**FIGURE 4:**
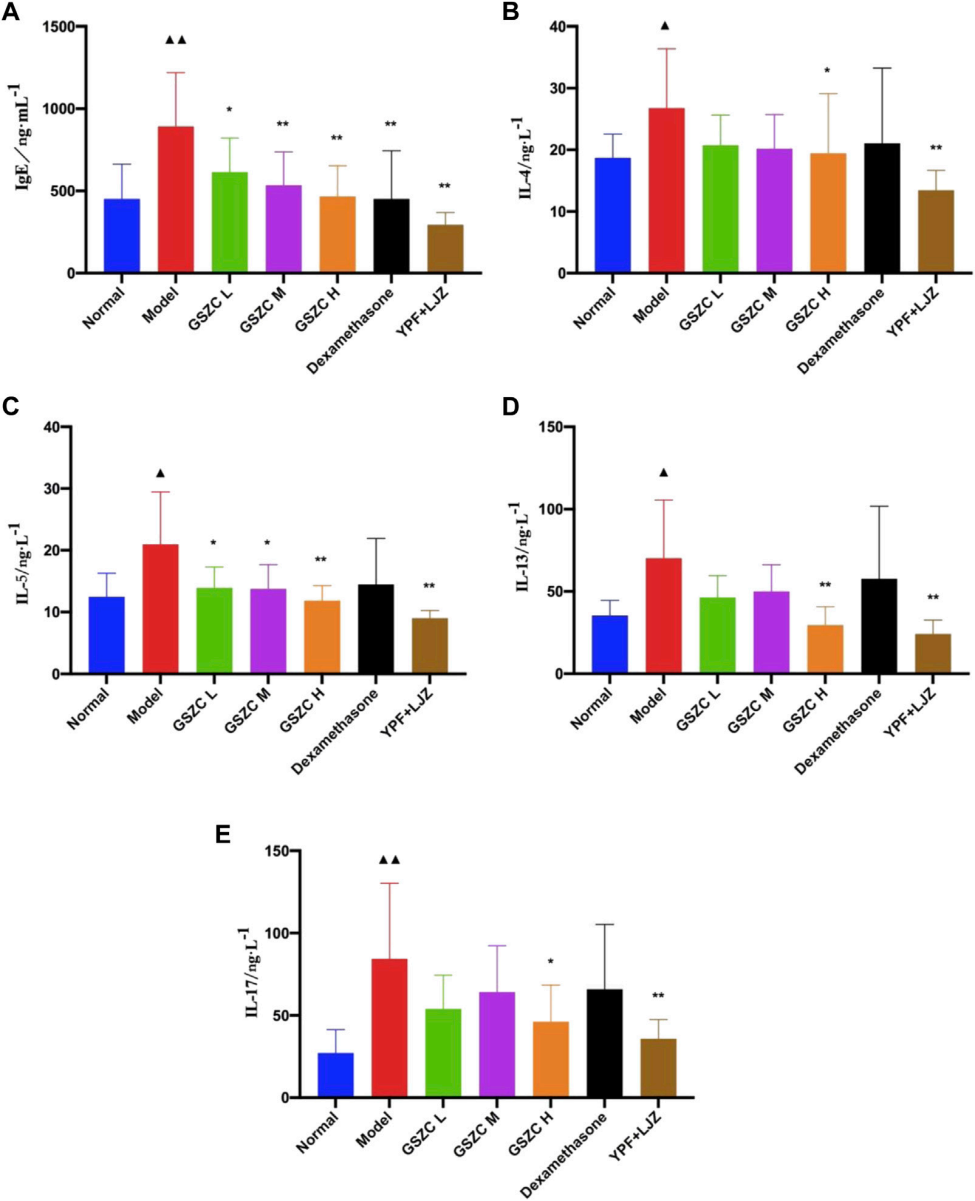
Effects of GSZC granules on serum IgE **(A)**, IL-4 **(B)**, IL-5 **(C)**, IL-13 **(D)**, IL-17 **(E)** in rats suffering from asthma. Values are the mean ± SD, n = 10 for each group. ^▲^ *p* < .05, ^▲▲^ *p* < .01 *versus* the normal group, ^*^ *p* < .05, ^**^ *p* < .01 *versus* the model group.

The model group showed a significant (*p* < .05 or *p* < .01) increase in serum levels of IgE, IL-4, IL-5, IL-13, and IL-17 compared with those in the normal group. A significant (*p* < .05 or *p* < .01) reduction in the IgE level was noted in all GSZC-granules groups, DEX group, and YPF + LJZ group. Levels of IgE and IL-4 were reduced significantly (*p* < .05 or *p* < .01) in the GSZC-H group and YPF + LJZ group compared with those in the model group. A significant (*p* < .05 or *p* < .01) reduction in the IL-5 level was noted in all GSZC-granules groups and YPF + LJZ group compared with those in the model group. A significant (*p* < .05 or *p* < .01) reduction in levels of IL-13 and IL-17 was documented in the GSZC-H group and YPF + LJZ group compared with those in the model group (Figure 4).

### 3.3 Effects of GSZC granules on %EOS and WBC count in the BALF of rats with asthma

**FIGURE 5:**
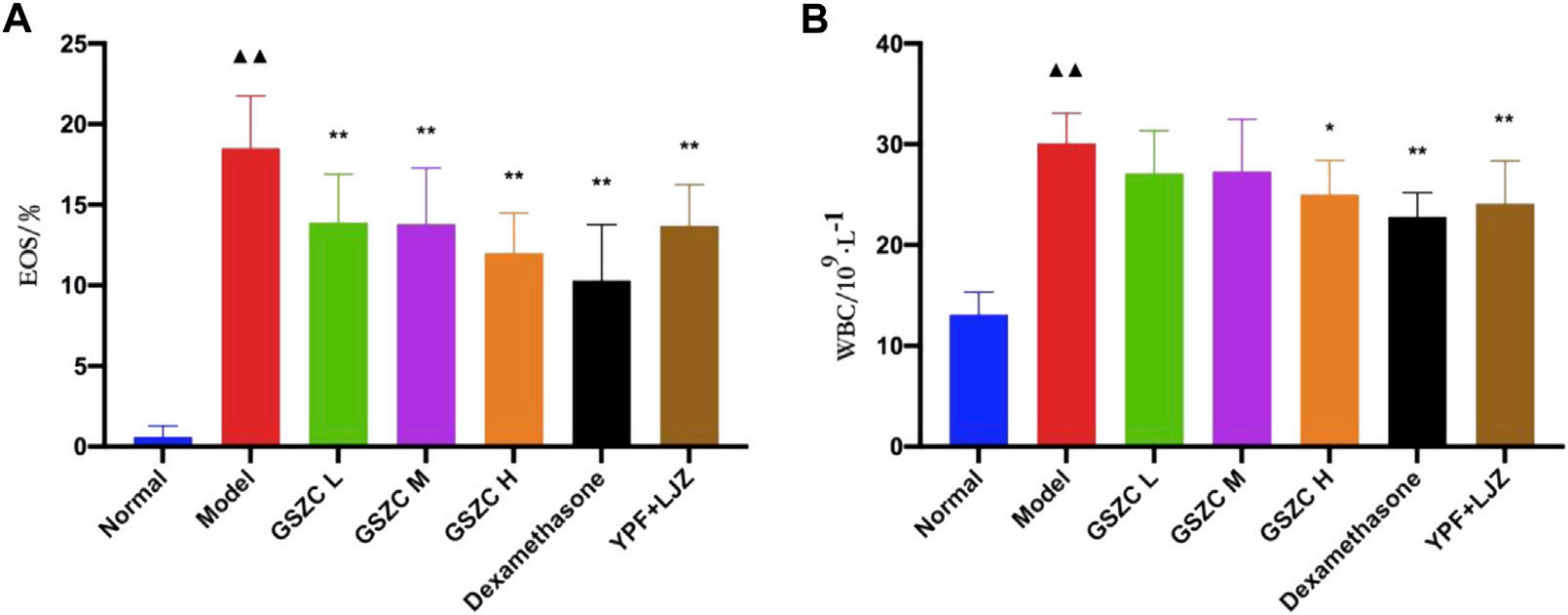
Effects of GSZC granules on EOS **(A)**, WBC **(B)** in BALF in rats suffering from asthma. Values are the mean ± SD, n = 10 for each group. ^▲▲^ *p* < .01 *versus* the normal group, ^*^ *p* < .05, ^**^ *p* < .01 *versus* the model group.

We discovered that %EOS and the WBC count was increased significantly (*p* < .01) in the BALF of rats in the model group as compared with those in the normal group. Additionally, %EOS was decreased significantly (*p* < .01) in each treatment group. The WBC count was decreased significantly (*p* < .01) in the GSZC-H group, DEX group, and YPF + LJZ group compared with that in the model group (Figure 5).

### 3.4 Effects of GSZC granules on histopathology in rat lungs

The lung tissues of rats in the normal group had normal bronchial walls. There was no significant necrosis/degeneration of cells or the mucosal epithelium. A modestly thick smooth-muscle layer and orderly arrangement were documented. Lung tissue from the model group exhibited thickening of bronchial walls, luminal stenosis, and degeneration of the mucosal epithelium with occasional necrosis and exfoliation. Hyperplasia of fibrous tissue was noted in the submucosa and lung interstitium, as was collagen deposition, hypertrophy and disarrangement of smooth muscle, and infiltration by many inflammatory cells.

**FIGURE 6:**
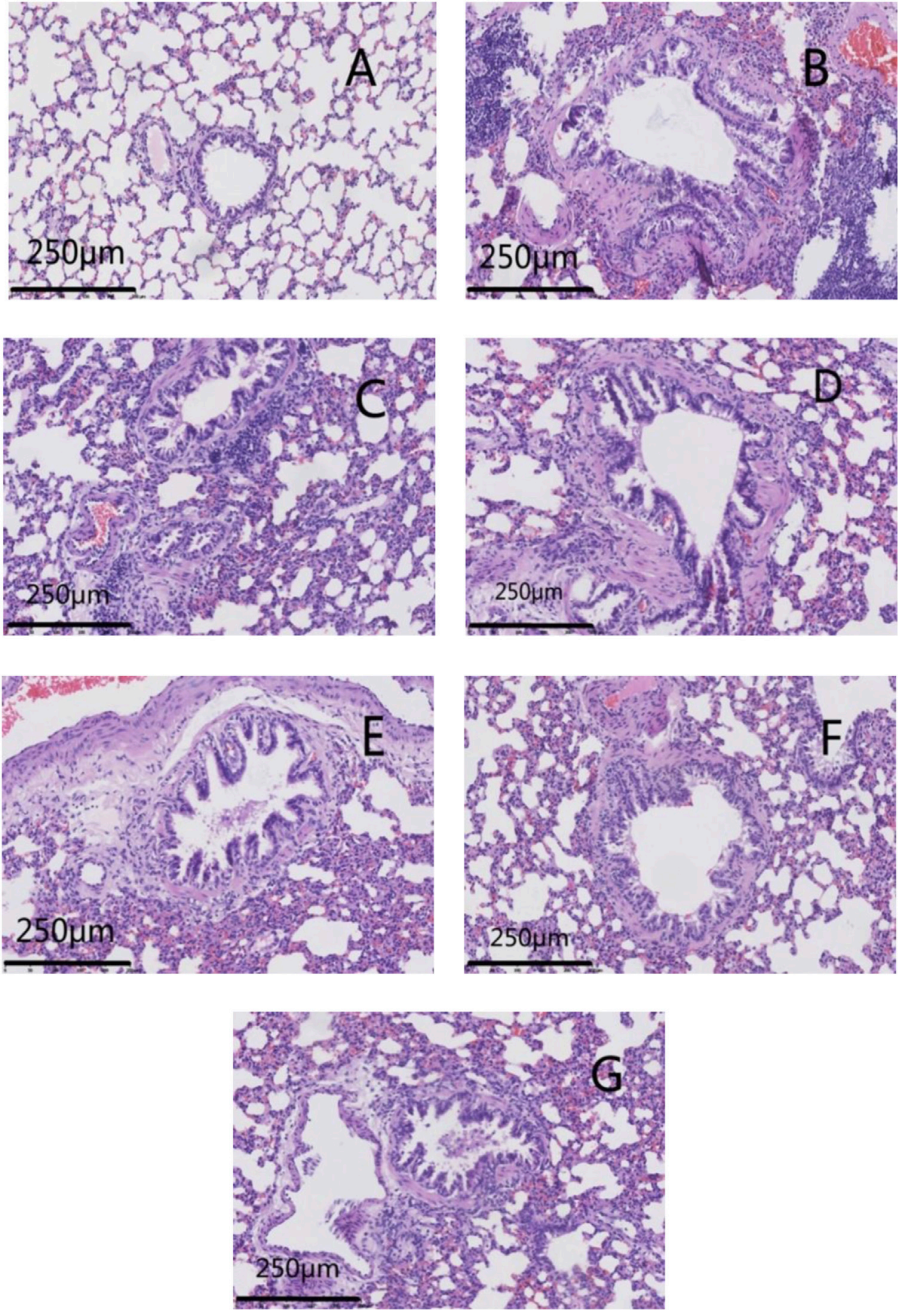
Effects of GSZC granules on the histopathology of lungs in rats suffering from asthma. Micrographs represent H&E staining in lung tissues. **(A)** is the normal group, **(B)** is the model group, **(C)** is the GSZC low dosage group, **(D)** is the GSZC moderate dosage group, **(E)** is the GSZC high dosage group, **(F)** is the Dexamethasone acetate group, **(G)** is the Yupingfeng granules + Liujunzi pills group, respectively. Scale bar: 250 μm. Magnification: 200×.

Lung tissue from rats in GSZC-M and GSZC-H groups revealed mild thickening of bronchial walls, slight hyperplasia of fibrous tissue in the submucosa and smooth muscle, with infiltration of many inflammatory cells. In the GSZC-L group, moderate thickening of bronchial walls, slight luminal stenosis, no necrosis, and slight hyperplasia of fibrous tissue were found in the submucosa. Also, collagen deposition, moderate hyperplasia of smooth muscle, slight hyperplasia of fibrous tissue in the lung interstitium, with infiltration by a moderate number of inflammatory cells, were documented. Lung tissue from rats in the DEX group exhibited slight thickening in the bronchial walls, slight hyperplasia in smooth muscle, and infiltration by a small number of inflammatory cells. (Figure 6).

**FIGURE 7:**
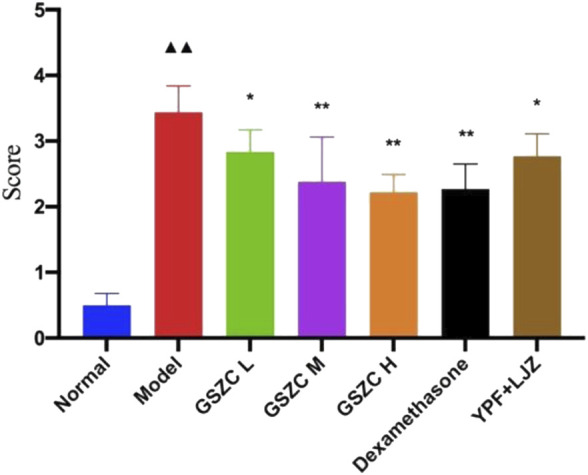
Effects of GSZC granules on lung histopathological score in rats suffering from asthma. Values are the mean ± SD, n = 6 for each group. ^▲▲^ *p* < .01 *versus* the normal group, ^*^ *p* < .05, ^**^ *p* < .01 *versus* the model group.

Lung tissue from rats in the YPF + LJZ group exhibited slight thickening in bronchial walls, slight hyperplasia in smooth muscle, with infiltration by a moderate number of inflammatory cells (Figure 6). The lung histopathological score from rats in the model group increased significantly (*p* < .01) compared with that in the normal group. Pathologic damage was alleviated, and the lung histopathological score decreased significantly (*p <*05 or *p* < .01) in each of the GSZC-granules groups, DEX group, and YPF + LJZ group (Figure 7).

### 3.5 Effects of GSZC granules on mRNA expression of MUC5AC and EGFR in the lung tissue of rats with asthma

**FIGURE 8:**
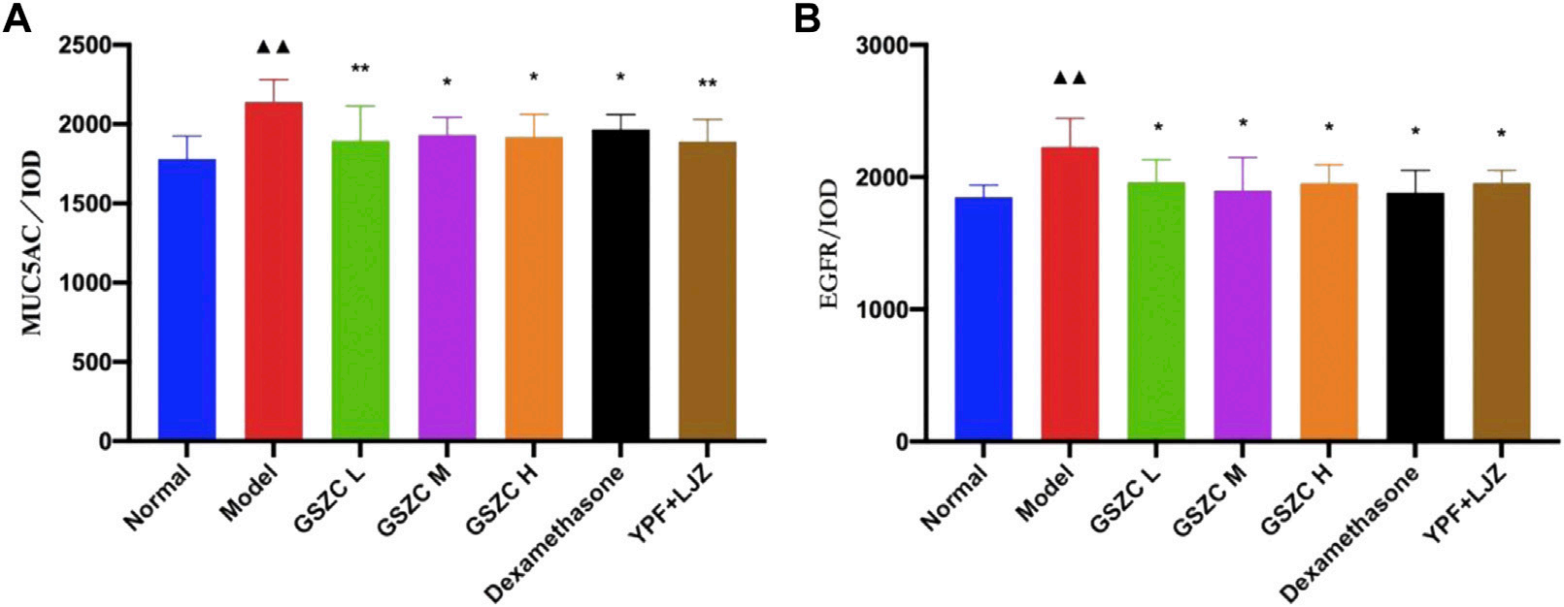
Effects of GSZC granules on lung MUC5AC **(A)**, EGFR **(B)** mRNA expression in rats suffering from asthma. Values are the mean ± SD, n = 6 for each group. ^▲▲^ *p* < .01 *versus* the normal group, ^**^ *p* < .01 *versus* the model group.

mRNA expression of MUC5AC and EGFR was increased significantly (*p* < .01) in the model group compared with that in the normal group. Significantly (*p* < .01) reduced mRNA expression of MUC5AC and EGFR was noted in the GSZC-H group, DEX group, and YPF + LJZ group compared with that in the model group (Figure 8).

### 3.6 Effects of GSZC granules on protein expression of MUC5AC and EGFR in the lung tissue of rats with asthma

**FIGURE 9:**
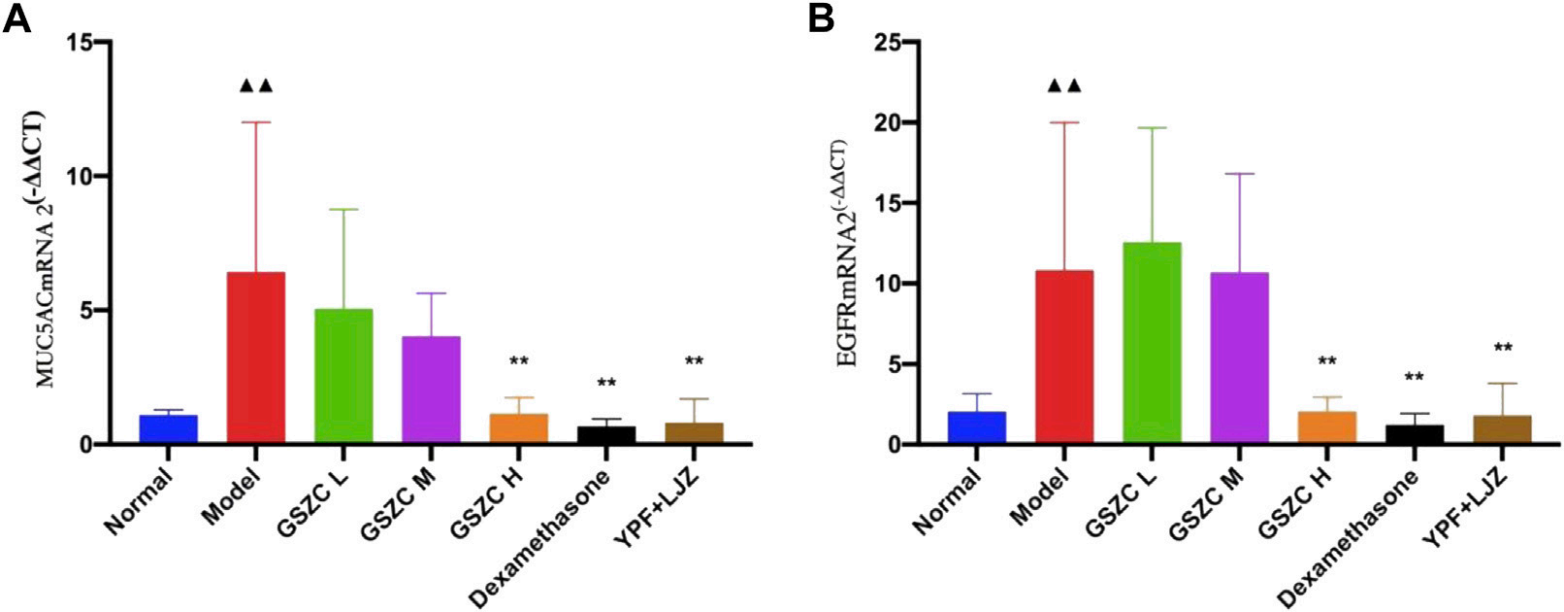
Effects of GSZC granules on lung MUC5AC **(A)**, EGFR **(B)** protein expression in rats suffering from asthma. Values are the mean ± SD, n = 6 for each group. ^▲▲^ *p* < .01 *versus* the normal group, ^*^ *p* < .05, ^**^ *p* < .01 *versus* the model group.

**FIGURE 10:**
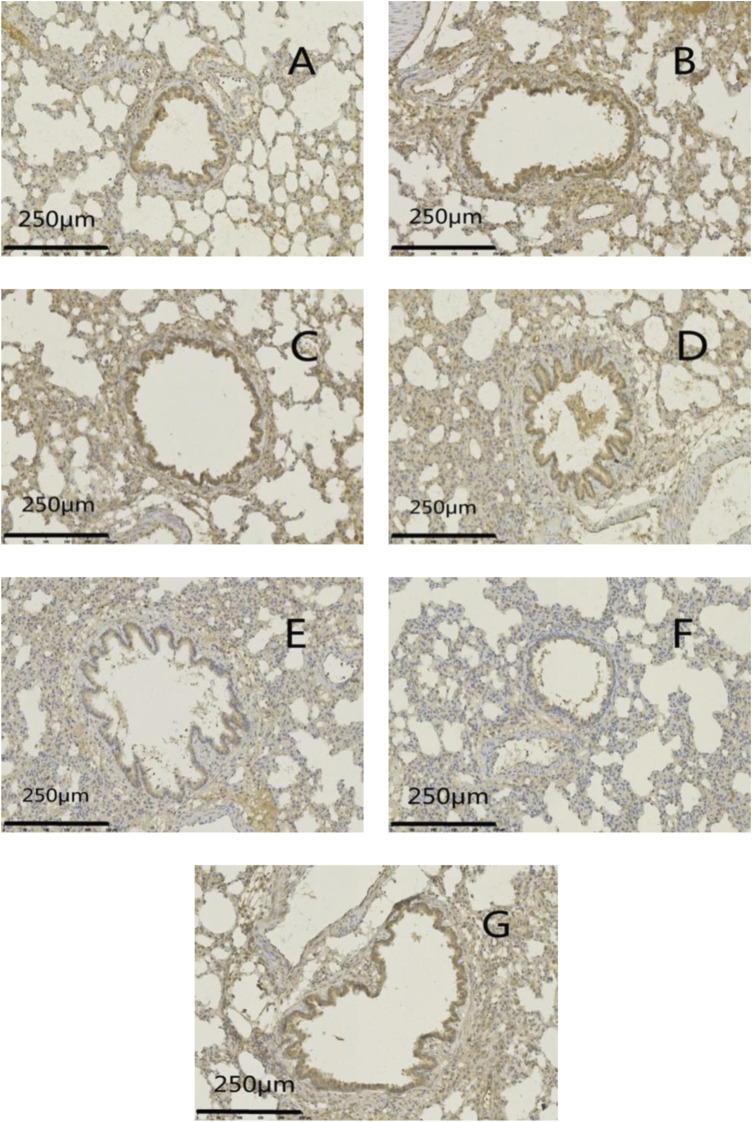
Effects of GSZC granules on lung MUC5AC protein expression in rats suffering from asthma. **(A)** is the normal group, **(B)** is the model group, **(C)** is the GSZC low dosage group, **(D)** is the GSZC moderate dosage group, **(E)** is the GSZC high dosage group, **(F)** is the Dexamethasone acetate group, **(G)** is the Yupingfeng granules + Liujunzi pills group, respectively. Scale bar: 250 μm. Magnification: 200×.

**FIGURE 11:**
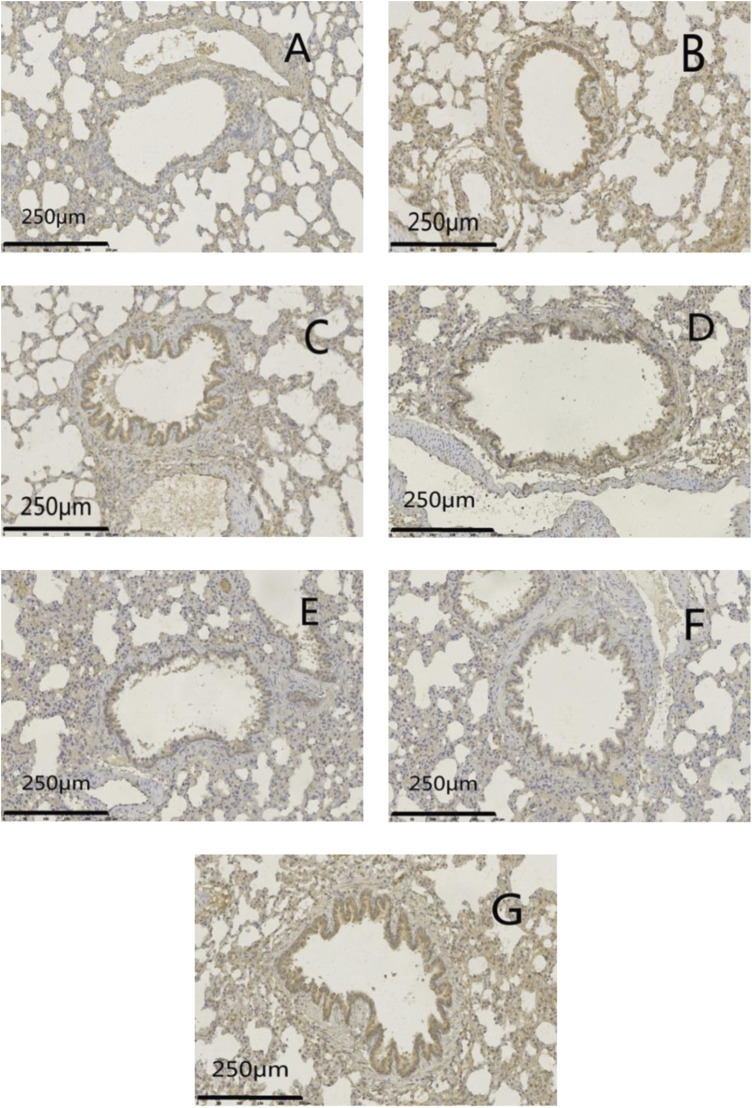
Effects of GSZC granules on lung EGFR protein expression in rats suffering from asthma. **(A-G)** were normal group, model group, GSZC low dosage group, GSZC moderate dosage group, GSZC high dosage group, Dexamethasone acetate group, Yupingfeng granules + Liujunzi pills group, respectively. Scale bars: 250 μm, magnification: 200×.

Protein expression of MUC5AC and EGFR was increased significantly (*p* < .01) in the model group compared with that in the normal group. Protein expression of MUC5AC and EGFR was reduced significantly (*p <*05 or *p* < .01) in administration groups compared with that in the model group (Figures 9–11).

## 4 Discussion

Use of an asthma model in rats has increased gradually in recent years, with Brown Norway, Sprague–Dawley, and Wistar rats being used most commonly. Brown Norway rats are used commonly (Ramos-Barbón et al., 2004; Zhou et al., 2017) because asthma symptoms can be induced sensitively and readily, and the immune response mediated by IgE can be induced (characterized by bronchial hyperresponsiveness and inflammation). However, Brown Norway rats have limited availability, are expensive, and can perish readily. Sprague–Dawley rats are relatively inexpensive, readily available, and have biological characteristics similar to those of humans, so they were used in this research.

Initially, we referred to the modeling method of Li and colleagues (Li, 2014). They injected 10% OVA/Al(OH)_3_ and inactivated pertussis toxoid at five time points, but eventually the dry powder of Al(OH)_3_ was replaced with a gel. Study has shown that inactivated pertussis toxoid is hard to obtain but has a therapeutic effect upon allergic asthma (Zhang, 2011). Thus, Liu and colleagues (Liu et al., 2017), Ji and coworkers (Ji et al., 2006), and Deng and collaborators (Deng et al., 2017) sensitized rats by injection of OVA solution (10 mg) with Al(OH)_3_ gel (100 mg) at three time points, and challenged with aerosolized 1% OVA to establish an asthma model.

Bronchial hyperreactivity is one of the objective indicators to judge if an asthma model is successful (Zhang and Li, 2011). The 2016 edition of the Global Initiative for Asthma (Sun and Chen, 2016) also uses lung function as an indicator of risk prediction and evaluation for asthma patients. In our study, the asthma model was deemed successful if airway reactivity was increased and C_dyn_ was decreased in the model group. Bronchial hyperresponsiveness is another main pathologic feature of asthma (Mandlik and Mandlik, 2020). Bronchial smooth muscle can undergo strong contractions and spasms, resulting in airway narrowing and an increase in R_I_, thereby inducing asthma. Production of cytokines such as IL-17, tumor necrosis factor (TNF)-α, and transforming growth factor (TGF)-β_1_ aggravate shrinking of bronchial smooth muscle, and induce bronchial hyperresponsiveness through the RhoA-ROCK (Ras homolog gene family, member A and its downstream effector Rho-associated protein kinase) pathway and calcium signaling pathways. R_I_ and C_dyn_ are used as indicators for evaluating asthma. GSZC granules was found to reduce R_I_, increase C_dyn_, and improve the bronchial hyperresponsiveness of rats, and the effect was similar to positive drugs.

Bronchial inflammation is the main pathogenesis of asthma (Feng et al., 2011). Inflammatory cells such as EOS and lymphocytes have key roles in asthma development. When asthma occurs, many inflammatory cells and cytokines are produced and released, and act together. WBCs are immune cells that have defensive and protective effects in the body, and inflammation occurs if their numbers increase. EOS are the final effector cells in allergic reactions. They can synthesize and secrete many proinflammatory factors, chemokines, and growth factors, which leads to shedding of epithelial cells, increased mucosal permeability, increased mucus secretion, thickening of bronchial smooth muscle, and destruction of the epithelial barrier in the lungs, thereby causing inflammation, hyperresponsiveness, and remodeling of the airways (Liu H. et al., 2019). Each dose of GSZC granules was shown to reduce the %EOS in BALF, and a high dose of GSZC granules could reduce the WBC count. In reducing the %EOS and WBC in BALF, effect of GSZC at high doses was more prominent, and the effect was comparable to positive drugs.

Asthma is a type-I hypersensitivity reaction mediated by IgE. Th2 cell-related cytokines can stimulate B cells to produce large amounts of IgE, promote activation of mast cells and increase the EOS number, thereby leading to airway inflammation. The main cytokines Th2 cells secrete are IL-4, IL-5, and IL-13. The most important cells in asthma are mast cells, EOS, and basophils (Kong et al., 2018). In general, IL-4 plays a key part in the differentiation of Th0 cells into Th2 cells and the initial sensitization of allergens. IL-5 is an important mediator that leads to an increase in the EOS number in the airway. It controls the growth, maturation, adhesion, secretion, and apoptosis of EOS (Chung et al., 2015). Th2 cells also act directly on epithelial cells and smooth muscle cells through IL-13 production. Therefore, IL-4, IL-5, and IL-13 are the critical effector cytokines in the Th2-related type-II immune response (Shin et al., 2014). Th17 cells are characterized by production of IL-17 and IL-6 (Chen et al., 2015), and IL-17 has a proinflammatory effect. Accumulating evidence suggests that IL-17 is involved in the histopathology of autoimmune diseases (including asthma), which can induce the recruitment of airway macrophages and increase their survival (Kaur et al., 2014). Th2/Th17 cell-associated cytokines have key roles in coordinating chronic airway inflammation by recruiting EOS, macrophages, and neutrophils, thereby promoting their survival and expanding airway inflammation. This inflammatory cycle can lead to shedding of epithelial cells, excessive secretion of mucus, and hypertrophy or contraction of smooth muscle, which are characteristic features of asthmatic airways (Lambrecht and Hammad, 2015; Alfardan et al., 2018). We showed that GSZC granules have similar effect to TCM-positive drug in terms of reducing serum levels of IgE, IL-4, IL-5, IL-13, and IL-17. The histopathology results stated in section 3.4 show that GSZC granules could reduce infiltration of inflammatory cells, improve pathologic damage, and reduce the Inflammation Score in the lung tissue of Sprague–Dawley rats, and the effect was comparable to positive drugs.

Mucus hypersecretion is a pathologic feature of airway inflammation. Mucus hypersecretion can lead to the obstruction, damage, and hyperresponsiveness of airways, as well as aggravate dyspnea, cause respiratory infections, and increase the incidence and mortality of asthma (Shi et al., 2017). Several factors (e.g., irritating gases and proinflammatory cytokines) can cause abnormal secretion of mucin in the airway epithelium, thereby leading to high mucus secretion. Clinical studies have shown that MUC5AC is the most abundant protein in the mucus of patients with asthma (Kim et al., 2020). IL-4, IL-13, and TNF-ɑ can lead to MUC5AC expression in bronchial epithelial cells, which can cause the proliferation of goblet cells and promote mucus production. IL-17 is also closely related to mucus secretion. Increased local secretion of IL-6 may be why IL-17 promotes MUC5AC expression in mucin. IL-17 can stimulate IL-6 secretion through activated mouse fibroblasts, stimulated human lung fibroblasts, and bronchial epithelial cells (Ma et al., 2018).

MUC5AC and MUC5B in airway mucus are the main gel-formed mucins (Chen et al., 2003). MUC5AC shows high expression in goblet cells of superficial airways (Curran and Cohn, 2010). The frequency of mucociliary beating is closely related to MUC5AC expression in inflamed airways (Oliviero et al., 2016; Nabissi et al., 2018). The EGFR is expressed only in the bronchial epithelium and glands in normal airways, but EGFR expression is seen in the bronchial smooth muscle and basement membrane of patients suffering from asthma (Warner and Knight, 2008). EGF, as a single-chain polypeptide with strong biological activity, can promote the differentiation and proliferation of epithelial cells, fibroblasts, and smooth muscle cells, and enhance epidermal growth and wound healing (Long, 2016). It acts mainly by binding to its receptor EGFR, which has a strong regulatory effect on airway epithelial cells and plays an important part in their repair and remodeling (Guo et al., 2012). Asthma patients express more EGFR in the airway epithelium and it has stronger functions than those observed in healthy people. EGF binds to the EGFR on the surface of damaged cells and forms a dimer, and activates related downstream cell-signaling pathways. This action generates a series of transduction cascades, accelerates the growth and differentiation of cells, and participates in repair of tissue damage. Repeated damage and repair accelerates the synthesis and deposition of the extracellular matrix, thereby aggravating airway remodeling (Chiba et al., 2010; Ding et al., 2014).

The MUC5AC/EGFR (Ramos-Barbón et al., 2004) signaling pathway plays a pivotal part in mucus secretion in the airway. The synthesis and secretion of mucin are strongly associated with the EGFR signaling pathway. Various factors activate the EGFR and further increase expression of mucin in the airway. For example, IL-13, TGF-α, and TNF-α can activate the EGFR. As a cytokine of Th2 cells, IL-13 is the central mediator of asthma. It can promote the metaplasia of goblet cells in inflammatory diseases. It activates the EGFR pathway, IL-13/signal transducer and activator of transcription-6 signaling pathway, calcium-activated chloride channels, and other pathways to promote expression of the gene and protein of MUC5AC (Jing et al., 2019). Protein and mRNA expression of MUC5AC and the EGFR in the model group was increased in the present study. GSZC granules could downregulate expression of IL-13, MUC5AC, as well as EGFR protein and mRNA in serum. We suggest that one of the mechanisms by which GSZC granules improve airway inflammation is intervention in the MUC5AC/EGFR signaling pathway, and inhibition of mucus hypersecretion in the airways of rats suffering from asthma.

## 5 Conclusion

GSZC granules could improve bronchial hyperresponsiveness, bronchial inflammation, and histopathologic damage in the lungs of rats suffering from asthma. This phenomenon may be related to its regulation of cytokine levels and the MUC5AC/EGFR signaling pathway.

## Data Availability

The original contributions presented in the study are included in the article/supplementary material, further inquiries can be directed to the corresponding authors.

## Abbreviations

OVA, ovalbumin; RI, airway resistance; Cdyn, dynamic compliance; Ig, immunoglobulin; IL, interleukin; ELISA, enzyme-linked immunosorbent assay; EOS, eosinophils; WBC, white blood cell; BALF, bronchoalveolar lavage fluid; EGFR, epidermal growth factor receptor; RT-qPCR, reverse transcription-quantitative polymerase chain reaction; TCM, traditional Chinese medicine; GSZC, Guishaozichuan; HPLC, high-performance liquid chromatography; H&E, hematoxylin and eosin; Th2, T helper type 2.
